# Should Arteriovenous Fistulas Be Ligated Electively after Successful Kidney Transplantation?: PRO

**DOI:** 10.34067/KID.0000000750

**Published:** 2025-12-23

**Authors:** Ulrika Hahn Lundström

**Affiliations:** Division of Renal Medicine, Department of Clinical Science, Intervention and Technology, CLINTEC, Karolinska Institutet and Karolinska University Hospital, Stockholm, Sweden

**Keywords:** cardiovascular disease, heart failure, hemodialysis access, kidney transplantation, vascular access

Ligation of the arteriovenous (AV) fistula should be considered in stable kidney transplant (KTx) recipients with persistent high-flow fistulas and well-functioning kidney grafts after the high-risk first post-transplant year. The AV fistula is the preferred access option for patients in hemodialysis, while how to handle functional AV fistulas in the KTx patient is debated. Cardiovascular disease is the main cause of mortality in KTx patients, and functional AV fistulas have been associated with unfavorable effects on heart and transplant function. At the same time, the effect of AV fistulas on hard end points such as kidney graft survival and patient mortality remains to be studied. A recent meta-analysis supported AV fistula ligation in transplant recipients on the basis of superior long-term cardiac function and improved kidney graft function.^1^ These studies and the improved kidney graft survival in recent years make the question of AV fistula ligation after successful kidney transplantation even more important.

## AV Cardiotoxicity and Heart Failure

The term AV access cardiotoxicity refers to a high cardiac output (CO) heart failure due to volume overload; the increased CO and circulatory changes with AV fistulas were described already decades ago.^2^ The CO increases due to the AV fistula flow, with decreased vascular resistance and increased venous return, as a compensatory response to maintain organ perfusion.^2^ This AV access cardiotoxicity is most pronounced in high-flow AV accesses, >1500 ml/min, with a strong correlation to CO.^2^ High-flow AV fistulas also cause pulmonary hypertension which, together with left ventricular (LV) hypertrophy, are both strong independent risk factors for heart failure and mortality in kidney patients.^2^ Already 6 months after AV fistula creation, a significant increase in CO and LV mass is described, and LV hypertrophy is seen in most incident dialysis patients.^2^ A functioning AV fistula when the patient undergoes KTx predicts *de novo* heart failure and constitutes a stronger risk factor for LV hypertrophy than time on dialysis, age, or eGFR.^3^ High-flow accesses also seem to influence distant organ perfusion by flow competition because of the strong relationship between access flow and CO.^2^ Decreased brain artery flow is a hemodynamic consequence of the competitive AV fistula and a possible reason for the cognitive impairment often seen in ESKD patients.^2^ Another example of possible flow competition is the left internal mammary grafts often used in aortocoronary bypass where ipsilateral upper arm AV fistulas should be avoided.^2^

## AV Fistula Ligation

AV fistula ligation is usually performed in high-flow AV fistulas, high-risk cardiovascular KTx patients, or for cosmetic reasons. Previously, observational studies have associated AV fistula ligation with improved cardiac function. Ligation is sometimes performed in patients with refractory heart failure, whereas routine AV fistula ligation has not been the case.^2^ This may change now since a randomized trial recently associated elective AV fistula ligation with significantly reduced LV mass, CO, and N-terminal pro-brain natriuretic peptide at 6 months.^4^ These prognostic data with improved heart function after KTx and AV fistula ligation are still unknown to most cardiologists, nephrologists, and access surgeons and are not described in current guidelines. Increased knowledge of the potential cardiotoxicity of nonused AV fistulas is key when treating KTx patients with heart failure or atrial flutter.

Nevertheless, the effect of AV fistula ligation on eGFR decline remains uncertain, whereas studies suggest both deterioration and improvement of eGFR after ligation.^2^ In retrospective studies, a preserved AV fistula is associated with an increased risk of future KTx graft loss and lower eGFR, while the ligation of a functioning AV fistula is associated with short-term eGFR decline.^5,6^ One could argue whether the retrospective design of these studies affects the comparability; if different KTx patients are advised to keep or ligate their AV fistula, there could be a risk of confounding by indication. Altogether, there is a need for further research, preferably with a prospective randomized design.^5^ Similarly, AV fistula creation is associated with reduced eGFR decline in several studies; however, when comparing the eGFR decline after AV fistula with peritoneal catheter placement, there were no significant differences, thus questioning any specific effect from AV fistulas on eGFR decline.^7^

In Europe, the 10-year KTx graft survival has improved to over 80% in recent years, which increases the importance of AV fistula ligation in KTx patients over time.^8^ Nevertheless, some argue against AV fistula ligation since some patients do experience KTx failure with the need to use their AV fistula for hemodialysis. There is a difference between AV fistula patency and the actual need to use the AV fistula for hemodialysis after KTx graft failure. In the literature, there are a few studies on AV fistula patency; an Italian study reported a 10-year 55% AV fistula patency; nonetheless, only 89/365 (24%) experienced KTx graft failure during follow-up, and 49/89 (55%) started hemodialysis with the AV fistula; in total, only 49/365 (13%) made use of their original AV fistula.^9^ A Slovenian study reported a 5-year 61% AV fistula patency, 127/626 (21%) experienced KTx graft failure during follow-up, 53/127 (40%) started hemodialysis, and in total only 53/626 (8%) used their original AV fistula.^6^ Interestingly, both studies had similar AV fistula failure rates, 3/4 spontaneous thrombosis and 1/4 underwent AV fistula ligation.^6,9^ Unfortunately, spontaneous thrombosis occurred in the less cardiotoxic low-flow AV fistulas, the lower arm fistulas or AV grafts, and more often in women. ^6^

Peritoneal dialysis (PD) is the suggested dialysis modality of choice in kidney patients with heart failure.^2^ A recent meta-analysis suggested PD as the preferred dialysis modality before KTx, with a lower risk for not just delayed graft function, but also superior long-term patient survival.^2^ Nevertheless, only a small minority of European kidney patients get access to home dialysis and PD before the KTx and a majority return to hemodialysis on KTx graft failure. This underlines the importance of increased access to multidisciplinary predialysis care to increase access to home dialysis and PD. The challenge in KTx patients is to balance cardiovascular hemodynamics and the cardiotoxic effect of AV fistulas on eGFR decline and the timing of KTx graft failure. The Kidney Failure Risk Equation has proved its utility, not just to predict KTx graft failure, but also as a risk-based AV access planning tool.^10^

In conclusion, AV fistulas should be considered for elective ligation in successful KTx recipients after the high-risk first post-transplant year to optimize long-term cardiac function, KTx graft, and patient survival. Currently, with the improved KTx graft survival, a remaining functional unused AV fistula in a KTx patient increases the risk of heart failure, lower eGFR, and KTx graft loss. In randomized studies, AV fistula ligation significantly improved cardiac function and heart failure. Factors to consider in the decision of AV fistula ligation in a KTx patient are the cardiotoxic effect of high-flow AV fistulas against the risk for future eGFR decline and KTx graft failure. There is a need for future research and updated guidelines to improve the care of our kidney patients.

## Supplementary Material
