# Supplementary material for: Should Arteriovenous Fistulas Be Ligated Electively after Successful Kidney Transplantation?: PRO
Source: Kidney360. 2025 Dec 23;7(8):1887–8. doi: 10.34067/KID.0000000750 (PMC13567842; doi:10.34067/KID.0000000750)
Supplement: Supplementary file 1 [file kidney360-7-1887-s001.pdf]

ASN American Society of Nephrology | JASN Journal of the American Society of Nephrology | KIDNEY360 Accessing Our World From Every Angle | CJASN Clinical Journal of the American Society of Nephrology

ASN Journal Disclosure Form

As per ASN journal policy, I have disclosed any financial relationships or commitments I have held in the past 36 months as included below. I have listed my Current Employer below to indicate there is a relationship requiring disclosure. If no relationship exists, my Current Employer is not listed.

U. Hahn Lundström reports the following:
Employer: Karolinska Institutet; Consultancy: Speaker and consultancy work for Baxter Medical and Fresenius Medical; Honoraria: Consultancy work for Vantive; and Advisory or Leadership Role: Baxter Medical; Attended Think Tank for predialysis education and increased self-dialysis.

I understand that the information above will be published within the journal article, if accepted, and that failure to comply and/or to accurately and completely report the potential financial conflicts of interest could lead to the following: 1) Prior to publication, article rejection, or 2) Post-publication, sanctions ranging from, but not limited to, issuing a correction, reporting the inaccurate information to the authors' institution, banning authors from submitting work to ASN journals for varying lengths of time, and/or retraction of the published work.

Name: Ulrika Hahn Lundström
Manuscript ID: K360-2025-000150R1
Manuscript Title: "Should AV fistulas be ligated electively after successful kidney transplantation: PRO"
Date of Completion: February 11, 2025
Disclosure Updated Date: February 11, 2025
